# Supplementary material for: Growth Hormone Secretory Capacity Is Associated with Cardiac Morphology and Function in Overweight and Obese Patients: A Controlled, Cross-Sectional Study
Source: Cells. 2022 Aug 4;11(15):2420. doi: 10.3390/cells11152420 (PMC9367721; doi:10.3390/cells11152420)
Supplement: Supplementary file 1 [file cells-11-02420-s001.zip › cells-1825214-supplementary.pdf]

Supplementary data

Table S1. General characteristics and anthropometric parameters of study population

	**Mean ± SD**
Age (years)	46.18 ± 12.78
BMI (kg/m2)	38.71 ± 7.88
Waist circumference (cm)	119.63 ± 17.42
Hip circumference (cm)	122.00 ± 14.38
WHR	0.96 ± 0.09
FM %	40.52 ± 6.68
TF (kg)	19.22 ± 6.10
TF %	38.90 ± 6.98
UFDI	1.87 ± 0.57
ASMM/weight	0.25±0.03

BMI = Body Mass Index; WHR = Waist-to-Hip Ratio; FM = Fat Mass; TF = Trunk Fat; UFDI = Upper body fat deposition index. ASMM = appendicular skeletal muscle mass

Table S2. Metabolic Parameters of our population.

	**Mean ± SD**
Fasting glucose (mg/dl)	97.73 ± 18.56
Basal Insulinemia (UI/L)	20.62 ± 19.49
Glycated hemoglobin %	5.68 ± 0.74
HOMA-IR	5.26 ± 5.67
Triglycerides (mg/dl)	137.82 ± 106.95
HDL (mg/dl)	49.09 ± 12.82
LDL (mg/dl)	118.95 ± 33.50
Total Cholesterol (mg/dl)	195.10 ± 36.99
CRP (mg/L)	0.65 ± 0.57
Systolic BP (mmHg)	129.87 ± 17.59
Diastolic BP (mmHg)	81.58 ± 11.73

HOMA-IR = HOmeostatic Model Assessment of Insulin Resistance; HDL = High Density Lipoproteins; LDL = Low Density Lipoproteins; CRP = C-Reactive Protein; BP = Blood Pressure.

Table S3. Echocardiographic Parameters of our population.

	**Mean ± SD**
EF %	64.79 ± 4.83
E/A	1.06 ± 0.26
cFS %	38.60 ± 3.50
EFT (mm)	8.30 ± 1.19
LVMI (g/m^2)	111.28 ± 25.39
IVS (mm)	10.85 ± 1.53
LVPW (mm)	10.11 ± 3.01
LV EDD (mm)	49.99 ± 4.73
LV ESD (mm)	30.68 ± 3.94
LA (mm)	28.69 ± 4.18

EF = Ejection Fraction; E/A = ratio between E wave and A wave; cFS = circumferential Fractional Shortening; EFT = Epicardial Fat Thickness; LVMI = Left Ventricular Mass index; IVS = Inter Ventricular Septal dimension; LVPW = Left Ventricular Posterior Wall; LV EDD = Left Ventricular End Diastolic Diameter; LV ESD = Left Ventricular End Systolic Diameter; LA = Left Atrium.
